# OGG1 Mutations and Risk of Female Breast Cancer: Meta-Analysis and Experimental Data

**DOI:** 10.1155/2015/690878

**Published:** 2015-05-19

**Authors:** Kashif Ali, Ishrat Mahjabeen, Maimoona Sabir, Humera Mehmood, Mahmood Akhtar Kayani

**Affiliations:** ^1^Cancer Genetics Laboratory, Department of Biosciences, COMSATS Institute of Information Technology, Park Road, Chak Shahzad, Islamabad 44000, Pakistan; ^2^Nuclear Medicine Oncology & Radiotherapy Institute (NORI), Islamabad 44000, Pakistan

## Abstract

In first part of this study association between OGG1 polymorphisms and breast cancer susceptibility was explored by meta-analysis. Second part of the study involved 925 subjects, used for mutational analysis of OGG1 gene using PCR-SSCP and sequencing. Fifteen mutations were observed, which included five intronic mutations, four splice site mutations, two 3′UTR mutations, three missense mutations, and a nonsense mutation. Significantly (*p* < 0.001) increased (~29 fold) breast cancer risk was associated with a splice site variant g.9800972T>G and 3′UTR variant g.9798848G>A. Among intronic mutations, highest (~15 fold) increase in breast cancer risk was associated with g.9793680G>A (*p* < 0.009). Similarly ~14-fold increased risk was associated with Val159Gly (*p* < 0.01), ~17-fold with Gly221Arg (*p* < 0.005), and ~18-fold with Ser326Cys (*p* < 0.004) in breast cancer patients compared with controls, whereas analysis of nonsense mutation showed that ~13-fold (*p* < 0.01) increased breast cancer risk was associated with Trp375STOP in patients compared to controls. In conclusion, a significant association was observed between OGG1 germ line mutations and breast cancer risk. These findings provide evidence that OGG1 may prove to be a good candidate of better diagnosis, treatment, and prevention of breast cancer.

## 1. Introduction

8-Oxoguanine DNA glycosylase 1 (OGG1) is an important protein in base excision repair (BER) pathway which plays a key role in maintaining genome integrity and preventing cancer development [1]. OGG1 is encoded by the OGG1 gene and is an important protein acting as a key enzyme in BER pathway. It initiates the process by recognizing and directly removing 8-hydroxy-2-deoxyguanine (8-OHdG) adducts from damaged DNA by releasing the modified base and generating an AP site [2]. The OGG1 gene is located in chromosome 3p26.2 and this region of genome has frequently been detected missing or deleted in various tumors, particularly lung, colon, stomach, kidney, oesophageal, prostate, and breast tumors, suggesting the loss of OGG1 function as a possible contributor to tumorigenesis and loss of heterozygosity of markers [3]. There are two major isoforms of human OGG1, that is, isoform ∞-OGG1 (345 amino acids) and isoform *β*-OGG1 (424 amino acids) proteins. The first 316 amino acids are common for both isoforms, while the C-termini vary considerably. OGG1 has two important domains; the OGG-N domain containing mitochondrial localization signal (MLS, position 9–26) partially contributes to the 8-oxoG-binding pocket and the HhH-GPD domain (a helix-hairpin-helix structural element followed by a Gly/Pro-rich loop and a conserved aspartic acid) containing nuclear localization signal (NLS, 335–342) provides both the catalytic and DNA-binding functions of the DNA glycosylase [4]. The human OGG1 protein structure reveals another highly conserved motif which corresponds to the helix-hairpin-helix (HhH) motif and is characteristic of the active site of endonuclease III family of DNA glycosylases/AP lyases [5]. Inactivation of the OGG1 gene may lead to a higher risk of cancer because cells with accumulated 8-OH-G adducts still retain the ability to proliferate and a substantial increase of spontaneous mutation frequencies has been clearly identified in the DNA of mutant mice, bearing transgenic gene when exposed to exogenous carcinogens or endogenous oxidative species [6]. These observations suggest that OGG1 acts as a major protein in pathway responsible for removal of 8-oxoG or 8-OH-G adducts [7].

OGG1 gene is highly polymorphic among humans and is also mutated in cancer cells. Epidemiologic studies have linked single nucleotide polymorphisms (SNP) in DNA glycosylase and BER core protein genes to human cancer risk including breast cancer [8, 9]. The OGG1 gene has at least twenty-five validated sequence variants that reportedly change amino acid of the protein but Ser326Cys (rs1052133) has been extensively investigated for its association with different types of cancer risk including esophageal [10], lung [11], stomach [12], thyroid [2], laryngeal [13], colorectal [14, 15], and pancreatic cancer [9]. The results about OGG1 polymorphisms are contradictory and further studies involving different populations are required. Present study is designed to observe the mutational spectrum of OGG1 and its association with different environmental, clinical, and histopathological parameter in breast cancer patients in Pakistani population. Initially a meta-analysis was performed involving previous studies and then the results were compared to obtain a clear picture about the role of OGG1 variations in breast cancer.

## 2. Materials and Methods

### 2.1. Search Strategy and Selection Criteria for Meta-Analysis

A comprehensive literature search was conducted using Pub Med database for all eligible studies (updated from January 2007 to November 2014) reporting OGG1 polymorphism/mutations, using the following search strategy: cancer, OGG1, polymorphisms, mutations, and genetic variations. There was no restriction on sample size, ethnicity of population, language, or type of report. All eligible studies were retrieved and checked for other relevant studies. The literature retrieval was performed in duplication by two independent reviewers. Studies were included only if they met the following criteria: (1) case-control studies which evaluated the association between OGG1 polymorphisms and cancer risk; (2) studies using DNA extracted from blood samples of cancer patients and also from healthy individuals used as controls for comparison; (3) studies using any of the mutation detection techniques (e.g., PCR-RFLP, PCR-SSCP, ARMS-PCR, and qRT-PCR arrays); (4) studies published as full articles in English.

A number of studies were excluded on the basis of the following points. (1) Studies using cancer cell lines, tumor samples, serum, or saliva samples were not included. (2) Review articles and previous meta-analysis were also not included. (3) Studies on diseases other than cancer were also excluded from present study.

### 2.2. Collection of Blood Samples

Present study was conducted with a prior approval from ethical committees of both COMSATS Institute of Information Technology Islamabad (CIIT) and collaborating hospitals. A total of 925 subjects were enrolled in present study including 530 female patients with histological confirmed breast cancer and 395 age and ethnicity matched cancer-free healthy female individuals as controls. Patients belonging to different areas of Pakistan were recruited from Nuclear Medicine, Oncology and Radiotherapy Institute (NORI) and Pakistan Institute of Medical Sciences (PIMS), Islamabad Pakistan, while controls were selected randomly and voluntarily from general population. The inclusion criterion for the controls was age and ethnicity matched healthy female individuals with absence of prior history of cancerous or precancerous lesions. Patients and controls suffering from any other familial disease (diabetes, blood pressure, and cardiovascular, renal, or hepatic impairment) were excluded from this study. After obtaining informed and written consent, each individual was personally interviewed using the specifically designed questionnaire. Information regarding age, age at menarche, menopausal status, menopausal age, family history, ethnic group, and tobacco use was collected from both patients and control individuals. Details regarding hormonal receptor status and histopathological findings were also recorded for clinical characterization of patients in first or follow-up meetings. Standard venipuncture was used to collect 5 mL of peripheral blood in EDTA containing tubes from patients and control individuals and was stored at −20°C until further use.

### 2.3. DNA Extraction and Polymerase Chain Reaction (PCR)

Genomic DNA was extracted from leucocytes, using standard phenol-chloroform extraction method as described by Baig et al. [16] with minor alterations. Freshly extracted DNA was quantified by spectrophotometry and yield gel electrophoresis and stored at −20°C till further processing. Human OGG1 exon sequence was taken from Ensemble. Primers were designed using primer 3 software and checked for their specificity using BLAST. Whole coding region including exon intron boundaries of approximately 60 bp sequence of OGG1 was investigated to identify novel, already reported, and any splice site variation. Each PCR reaction was performed in a 10 *μ*L reaction mixture containing 1 *μ*L of genomic DNA (approximately 50 ng) templates, 1 *μ*L (10 mM) of each primer, 1 *μ*L nuclease-free water, and 5 *μ*L PCR master mix (Thermo Scientific) containing 0.05 U/*μ*LTaq DNA polymerase, reaction buffer, 4 mM MgCl_2_, 0.4 mM of each dNTP. PCR conditions were initial melting step at 94°C for 5 min, 35 cycles each comprised of 94°C for 45 sec, exon specific annealing temperature for 1 min and 72°C for 1 min. It was followed by a final extension step at 72°C for 10 min and finally held at 4°C. 2 *μ*L of PCR products along with loading dye were electrophoresed on a 2% agarose gel and stained with ethidium bromide. 100 bp ladder was also loaded as standard for quantification of amount and confirmation of PCR product size.

### 2.4. Mutational Screening and Sequence Analysis

Single stranded conformational polymorphism (SSCP) assay was used for mutational analysis of PCR products. Samples with altered electrophoretic mobility were reamplified in a separate reaction and were analyzed by direct sequencing to confirm and characterize the nature of mutations/polymorphisms. Control (normal) samples were also sequenced along with cancerous samples to compare the sequencing results. DNA sequencing was carried out by MC lab (USA). Results of DNA sequencing were analyzed using BioEdit software (version 7.0.5) and Alamut visual interactive biosoftware (version 2.4-5).

### 2.5. Data Analysis

*χ* ^2^-test, Fisher's exact test, and Pearson correlation coefficient were used to analyze the differences in selected demographic variables, family history, smoking status, tumor types, tumor grades, ER/PR, and HER-2/*nue* status by using the Graph Pad Prism 5. Pearson's correlation coefficient was used to assess the correlations among the observed mutations and clinical and histopathological parameters. Missense mutations were analyzed in silico via Alamut biosoftware (version 2.4-5) for prediction of the pathogenicity caused by point mutations, PhyloP for conservation level of mutated nucleotides, and amino acids along with Grantham distance for physicochemical changes in amino acid structure.

## 3. Results

In first part of study a meta-analysis was performed to evaluate the association between OGG1 polymorphisms and cancer susceptibility especially as risk factor of breast cancer. Based on our search criteria, 152 studies relevant to the role of OGG1 mutations/polymorphisms on cancer/disease susceptibility were identified. 90 studies of total 152 were excluded on the basis of the following reasons.

(i) Five studies were review/meta-analysis, (ii) 8 studies were involving only general healthy population, (iii) 18 studies were involving OGG1 mutations in patients other than cancerous, for example, diabetes, cataract, endometriosis, and so forth, (iv) 14 studies used DNA samples from tissues other than blood samples of cancer patients, and (v) 45 studies were older than January 2007.

As a result, a total of 62 relevant studies (involving 32626 individuals including 14844 patients and 17782 healthy control individuals) met the inclusion criteria for the current meta-analysis. Among them, most of studies used PCR-RFLP (48) and other techniques (12) for detection of already reported one polymorphism Ser326Cys in cancer. Only two studies used techniques for the detection of reported as well as novel mutations in cancer, one involved high resolution melting (HRM) analysis and other one used PCR-SSCP. Of all eligible studies, the majority of studies were on head and neck, lung, and colorectal cancers whereas only 6 studies evaluated the OGG1 polymorphism in breast cancer. The majority of studies were from Caucasian (17), Chinese (16), and Indian (14) populations while only one study was from Pakistani population involving head and neck cancer patients. Moreover, only 4 of the available studies used patient sample size more than or equal to 500 and remaining 94% of studies used fewer number of patient samples. Only 3 studies recruited purely population based (PB) controls while all other studies involved hospital based (HB) controls. Findings of all previous studies investigated for this meta-analysis were contradictory regarding association of OGG1 polymorphisms to increased risk of cancer susceptibility. Out of selected 62 studies involving 32626 individuals (including 14844 patients and 17782 controls), thirty-five studies involving 19594 individuals (including 9071 patients and 10523 controls) concluded a contributory role of OGG1 polymorphism to different type of cancers while in twenty-six studies involving 12812 individuals (including 5663 patients and 7149 controls) no association of OGG1 polymorphism to cancer susceptibility was observed and only one study involving 220 individuals (including 110 patients and 110 controls) suggested negative or protective role of OGG1 polymorphism against cancer (Table 1). In summary, when all the eligible studies were pooled into the meta-analysis of OGG1 mutations, 60.1% individuals showed an association of OGG1 mutations with different types of cancers while 39.3% individuals showed no association and 0.7% individuals showed a negative or protective role of OGG1 mutations against cancer.

**Figure 1:**
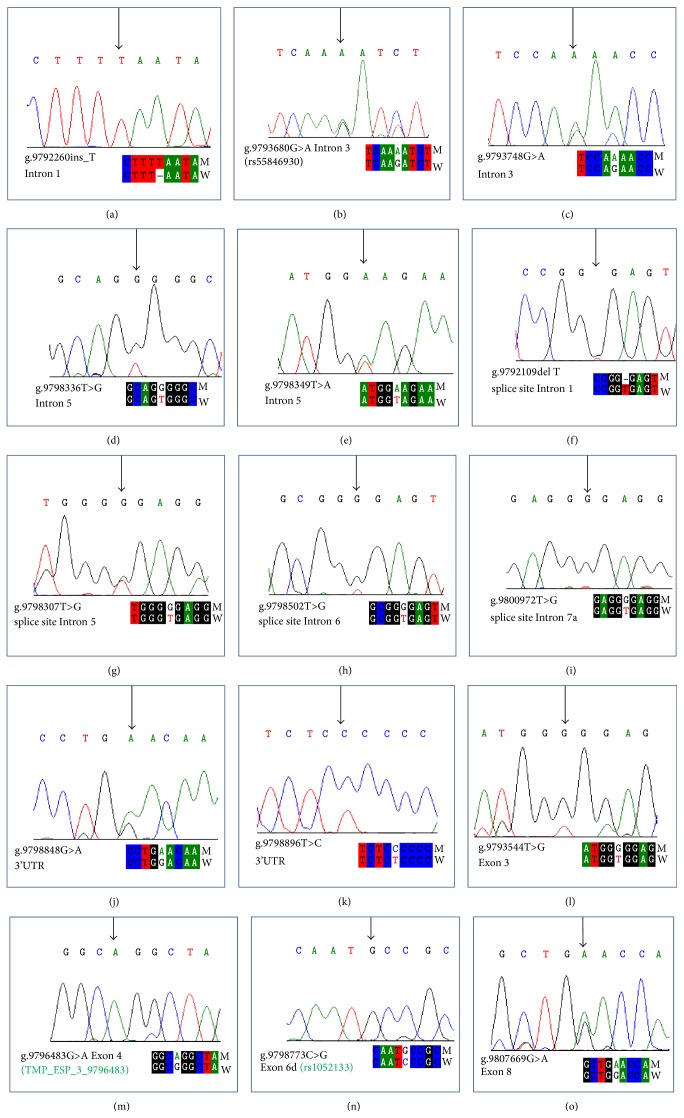
Sequencing electropherogram of polymorphisms of OGG1. (a), (b), (c), (d), and (e) are intronic mutations ((a) g.9792260 insertion of T in Intron 1, (b) g.9793680G>A (rs55846930) substitution in Intron 3, (c) g.9793748G>A substitution in Intron 3, (d) g.9798336T>G substitution in Intron 5, and (e) g.9798349T>A substitution in Intron 5). (f), (g), (h), and (i) are splice site mutations ((f) g.9792109 deletion of T at Splice site region of Intron 1, (g) g.9798307T>G substitution in splice site region of Intron 5, (h) g.9798502T>G substitution in splice site region of Intron 6, and (i) g.9800972T>G substitution in splice site region of Intron 7a). (j) and (k) are substitutions in 3′UTR ((j) g.9798848G>A substitution in 3′UTR (k) g.9798896T>C substitution in 3′UTR). (l), (m), (n), and (o) are missense mutations ((l) missense mutation Val159Gly showing g.9793544T>G substitution in Exon 3 resulting in change of codon from GTG to GGG encoding amino acid Valine instead of Glycine, (m) missense mutation Gly221Arg (TMP_ESP_3_9796483) showing g.9796483G>A substitution in Exon 4 resulting in change codon from GGG to AGG encoding the amino acid Glycine instead of Arginine, (n) missense mutation Ser326Cys (rs1052133) (CM993185) showing g.9798773C>G substitution in Exon 6d resulting in change of codon from TCC to TGC encoding the amino acid Serine instead of Cysteine, and (o) nonsense mutation Trp375STOP^*∗*^ showing g.9807669G>A substitutions in Exon 8 resulting in change of codon from TGG to TGA terminating the protein instead of encoding the Tryptophan amino acid).

Second part of present study involved 925 subjects including 530 breast cancer patients and 395 cancer-free healthy individuals as control used for mutational analysis of OGG1 gene. Mean age of patients and controls was calculated as 46.4 (±11.59) and 42.80 (±12.96) years, respectively (see Supplementary Tables 1 and 2 in Supplementary Material available online at http://dx.doi.org/10.1155/2015/690878). In present study, all (eight) exons of OGG1 were screened comprehensively for any novel or reported germline mutations involving SSCP followed by direct sequence analysis of suspected samples. Fifteen different types of mutations were observed, which included five intronic, four splice site, two 3′UTR, and four missense mutations. Among identified mutations, one intronic mutation (g.9793680G>A, rs55846930) and two missense mutations (Gly221Arg, TMP_ESP_3_9796483 and Ser326Cys, rs1052133) have already been reported while remaining twelve mutations were novel. Four novel mutations (g.9792260 insert_T; g.9793748G>A; g.9798336T>G; g.9798349T>A) were observed in intronic regions, four mutations (g.9792109delT, g.9798307T>G, g.9798502T>G & g.9800972T>G) were observed in splice site regions, two mutations (g.9798848G>A, g.9798896T>C) were observed in 3′UTR, one missense mutation (g.9793544T>G, Val159Gly) was observed in exon 3, and one nonsense mutation (g.9807669G>A, Trp375STOP) was observed in exon 8 (Figure 1).

Significantly increased breast cancer risk was found associated with different mutations when compared with controls (Table 2). Three intronic mutations (g.9792260 insert_T; g.9798336T>G; and g.9798349T>A) and one 3′UTR mutation (g.9798896T>C) were also detected in control samples but their frequency was significantly high in patients (*p* < 0.05). Significantly (*p* < 0.001) increased (~29 fold) breast cancer risk was found associated with a splice site variant g.9800972T>G (OR = 28.85, 95% CI = 3.87 to 207.7) and 3′UTR variant g.9798848G>A (OR = 29.20, 95% CI = 33.98 to 213.74). Among intronic mutations, highest (~15 fold) increase in breast cancer risk was associated with g.9793680G>A variation (OR = 14.65, 95% CI = 1.95 to 109.9; *p* < 0.009). Similar trend was observed in all detected missense mutations in breast cancer patients when compared with controls and ~14-fold increased risk was associated with Val159Gly (OR = 13.68, 95% CI = 1.82 to 102.9; *p* < 0.01), ~17-fold with Gly221Arg (OR = 16.85, 95% CI = 2.26 to 125.53; *p* < 0.005), and ~18-fold with Ser326Cys (OR = 18.45, 95% CI = 2.49 to 136.99; *p* < 0.004) in breast cancer patients compared with controls, whereas analysis of nonsense mutation showed that ~13-fold (OR = 12.90, 95% CI = 1.71 to 97.28; *p* < 0.01) increased breast cancer risk was associated with Trp375STOP in patients compared to controls.

**Figure 2:**
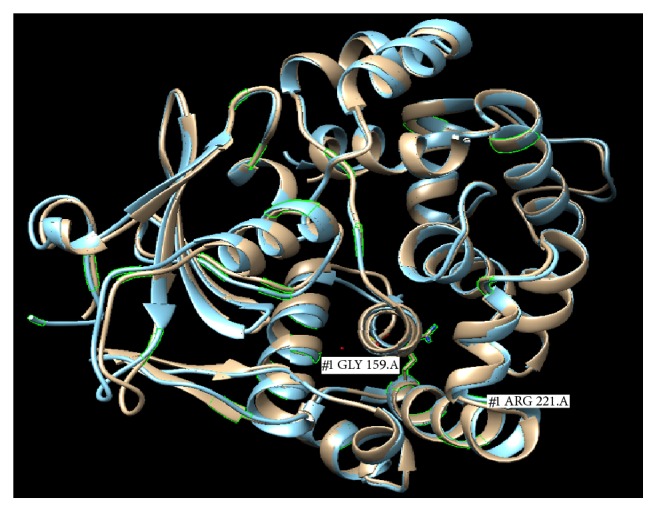
Superimposed protein structure of mutated OGG1 with its wild type. Wild-type OGG1 protein (grey) and mutated OGG1 protein (blue). Superimposed structure of mutated OGG1 protein showing the location of two observed mutations, Val159Gly and Gly221Arg. Wild-type protein model obtained from protein data bank. Structure was predicted using automated Swiss model. Two structures, wild and mutated, were aligned using UCSF chimera software.

Missense mutations Val159Gly, Gly221Arg, and Ser326Cys were observed in protein domains HhH-GPD and 8-oxoguanine DNA-glycosylase (Supplementary Table 3). Missense and nonsense mutations were also analyzed via Alamut biosoftware (version 2.4.5) to check the conservation levels of mutated nucleotides and amino acids along with in silico predictions about Align GVGD score, Grantham distance, SIFT score and Mutation Taster (Table 3). Mutation Taster predicted two missense mutations (Val159Gly and Gly221Arg) and one nonsense mutation (Trp375STOP) as potentially disease causing (*p* = 1.0). Greater physiochemical difference in protein structure was predicted in case of nonsense mutation Trp375STOP that resulted in truncated protein chain due to replacement of a moderately conserved amino acid Tryptophan with a stop codon (Grantham distance = 170). Protein modeling of two detected mutations (Val159Gly, Gly221Arg) of OGG1 and comparison with wild-type OGG1 protein has concluded that no major conformational change occurs due to these mutations while one nonsense mutation (Trp375STOP) resulted in truncation of protein (Figure 2).

Association of observed mutations was also correlated with different clinicopathological parameters including family history, menopausal age, and HER-2*/nue* and ER/PR status. Frequency of OGG1 mutations was observed to be significantly higher in patients with invasive ductal carcinoma (*p* < 0.0001), negative ER (*p* < 0.001), and negative PR status (*p* < 0.01). All observed OGG1 mutations were found significantly correlated with tumor types (*r* = −0.333^*∗∗∗*^; *p* < 0.0001), ER status (*r* = 0.739^*∗∗*^; *p* < 0.001), and PR status (*r* = −0.155^*∗*^; *p* < 0.01) of breast cancer patients but a nonsignificant correlation was observed between all mutations and HER-2/*neu* status (*r* = 0.318, *p* = 0.12) of breast cancer patients (Table 4). As shown in Table 5, significantly increased breast cancer risk was associated with an intronic (g.9793680G>A, *p* < 0.03), a splice site (g.9798502T>G, *p* < 0.03), and a missense (Ser326Cys, *p* < 0.009) mutation in patients with family history as compared to controls. Correlations between frequency of OGG1 mutations and menopausal age of breast cancer patients (Table 5) revealed that frequencies of three intronic mutations (g.9792260 ins_T; g.9793680G>A; and g.9798349T>A), two splice site mutations (g.9792109delT and g.9800972T>G), two 3′UTR mutations (9798848G>A and g.9798896T>C), and one missense mutation (Ser326Cys) were significantly higher (*p* < 0.05) in patients with earlier menopause (≤50 years) compared to controls and patients with late menopause (>50 years).  Table 6 showed association of OGG1 mutations with smoking status of patients and controls. Statistically significant (*p* < 0.05) association of OGG1 mutations (Ser326Cys, g.9792109delT, g.9800972T>G, g.9792260 ins_T, and g.9798848G>A) was observed with patients having smoking history compared to patients and controls with no smoking history.

## 4. Discussion

OGG1 is an important gene of BER pathway which encodes the enzyme responsible for the excision of 8-oxoguanine (8-oxoG), a mutagenic base byproduct which occurs as a result of exposure to reactive oxygen species (ROS) [11]. In first part of study a meta-analysis was designed to explore the association between OGG1 polymorphisms and breast carcinogenesis. Results of current meta-analysis revealed that 60 out of 62 selected studies focused only on OGG1 mutation (Ser326Cys) for its role in carcinogenesis and the majority of studies (60%) concluded association of this mutation with different cancers (Table 1). But mutations other than Ser326Cys, in the same domain or other domains of OGG1 singly or in combination may also be important in initiation and development of cancer as reported by Mahjabeen et al. [13]. Moreover studies involving relatively larger population for exploration of different OGG1 mutations (novel as well as reported) in relation to other clinicohistopathological parameters may also be needed for their role in cancer development.

Second part of present study is designed to screen all intronic and exonic regions of OGG1 gene in 925 individuals including 530 breast cancer patients and 395 controls using PCR-SSCP followed by sequencing. A total of fifteen mutations were identified in patients and in some control individuals. Eleven mutations were observed in different noncoding regions of OGG1 gene including five mutations in intronic regions, four mutations in donor splice site, and two mutations in 3′UTR regions. Among these, 12 mutations were novel and three were already reported (rs55846930, TMP_ESP_3_9796483, and rs1052133). Frequencies of these observed spice site mutations were found significantly higher in patients as compared to control individuals suggesting their association with breast carcinogenesis. Observed mutations were also analyzed by Alamut biosoftware (version 2.4-5) which predicted that skip of Exons 1, 5, 6, and 7 is very likely as mutations were observed in donor splice site areas of respective exons. Since splice site regions in a gene are involved in the processing of precursor mRNA into mature mRNA and deletion, insertion, or any substitution in the splice sites results in immature mRNA which may have one or more introns in it, leading to the production of aberrant proteins [17]. So mutations in these regions may be very crucial for cellular functioning.

In addition to these, three missense mutations (Val159Gly, Gly221Arg, and Ser326Cys) and a nonsense mutation (Trp375STOP) were also found significantly higher in breast cancer patients compared to control individuals suggesting their association with breast carcinogenesis. Among these, two missense mutations (Val159Gly in Exon 3 and Gly221Arg in Exon 4) were found in the HhH-GPD domain. HhH-GPD domain of OGG1 is much important as it performs the catalytic as well as DNA-binding functions of the DNA glycosylase so mutations in this domain might be pathogenic [18]. In this study another missense mutation, Ser326Cys, was also observed mainly as homozygous genotype. This mutation has already been extensively investigated and found to be associated in different types of cancers [9, 19]. Ser326Cys variant is located in 8-oxoguanine DNA-glycosylase domain, which is involved in DNA glycosylase activity of OGG1 protein [4]. Cells with Ser326Cys mutation in homozygous condition are reported to be much deficient in the repair of oxidative DNA damage especially when they are under excessive oxidative stress [20]. In addition to these, a nonsense mutation Trp375STOP was also observed in the C-terminus of *β* isoform of OGG1 protein resulting in truncation of protein which might compromise the proper functioning of OGG1 protein. Function of this specific region of C-terminus of *β* isoform of OGG1 is still not perfectly clear as it has been least investigated, whereas presence of long coiled tail, spanning a transmembrane domain in the C-terminus of *β* isoform of OGG1 protein, suggests its clear role in anchoring the protein in membranous structures [4].

Missense and nonsense mutations observed in this study were analyzed via Alamut biosoftware (version 2.4-5) and observed that missense mutations especially of highly conserved nucleotides (g.9793544T>G) and conserved amino acids (Val159Gly; Gly221Arg; and Trp375STOP) have shown some deleterious, potentially disease causing effects resulting physiochemical alterations in structure of amino acids. In silico predictions about mutations using PolyPhen-2 [21], SIFT [22], and Mutation Taster [23] software have previously been considered an important tool in exploration of possible effects of mutations and similar results were achieved by Alamut software in this study.

OGG1 mutation frequencies were also correlated with different clinicopathological parameters and significant findings were observed. Higher mutation frequencies were found to be associated with invasive ductal carcinoma, family history of cancer, early menopause, smoking history, and negative ER, PR, and HER-2/*neu* status which have been reported to contribute in breast cancer development in Pakistani populations [24, 25] and worldwide [25, 26]. Use of tobacco has been considered a well-known environmental risk factor of various cancers. Reactive oxygen species present in tobacco smoke produce 8-hydroxyguanine (8OH-G), which may cause oxidative DNA damage. The OGG1 protein is in front line of the cellular defense against oxidative DNA damage and to repair the 8-oxoG DNA adducts [27]. Decreased repair activity for removal of 8-hydroxyguanine adducts has been observed by homozygous mutant hOGG1 (Cys326Ser) protein [28].

## 5. Conclusion

In conclusion, we have observed a significant association of germ line mutations of OGG1 with breast cancer in Pakistani population in this study. Splice site, 3′UTR, missense, and nonsense mutations in highly conserved and functionally important domains of OGG1 protein alone or in combination with other genes of the BER pathway may contribute in the process of breast carcinogenesis, each adding a small effect on the overall cancer risk in Pakistani population. Moreover, in line with previous findings, inhibited or reduced DNA repair and enzymatic activities of OGG1 protein may potentially sensitize the tumour cells to therapeutic agents, making OGG1 an attractive molecular target in the treatment of cancer. These molecular and epidemiological findings provide evidence that OGG1, a DNA repairing gene, could prove to be a good candidate of better diagnosis, treatment, and prevention of breast cancer.

## Supplementary Material

In Supplementary material, supplementary Table 1 contained the detailed information regarding the demographic characteristics of patients and controls of sampled population. Significantly increased risk of breast cancer was found associated in patients with family history of cancer and similar trend was found in case age of menopause. Supplementary Table 1 also showed that significantly higher percentage of breast cancer patients were smokers as compared to controls. Supplementary Table 2 contained the information regarding the histopathological parameters of study cohort and majority of breast cancer patients were with invasive ductal carcinoma with grade-II and negative ER, PR and HER-2/neu status. Supplementary Table 3 represents the details of mutations observed in the OGG1 gene in breast cancer patients along with possible consequences of these changes at protein level.

## Figures and Tables

**Table 1:** Literature search in PubMed database for all eligible studies reporting OGG1 polymorphism.

Previous studies with OGG1 germ line mutation analysis								
Author and year	Population	Cancer type	Sample size		Source of control	Techniques	Mutations	Cancer risk association
	(Location and ethnicity)		Patients	Controls				
Arizono et al., 2008 [29]	Asian, Japanese	Bladder cancer	251	251	HB	PCR-RFLP	Ser326Cys	Associated
Bose et al., 2013 [30]	Asian, Indian	Liver cancer	214	210	HB	PCR-RFLP	Ser326Cys	Associated
Canbay et al., 2010 [31]	Caucasian, Turkish	Gastric cancer	40	247	HB/PB	PCR-RFLP	Ser326Cys	Associated
Canbay et al., 2011 [14]	Caucasian, Turkish	Colorectal cancer	79	247	HB	PCR-RFLP	Ser326Cys	Associated
Chang et al., 2009 [32]	Latino-African Americans	Lung cancer	368	579	HB	PCR-RFLP	Ser326Cys	Not associated
Chen et al., 2010 [33]	Asian, Chinese	Pterygium	83	206	HB	TaqMan assays	Ser326Cys	Not associated
Chen et al., 2011 [34]	Asian, Chinese	Breast cancer	518	777	HB	HRM analysis	c.-18G>T, c.-23A>G,	Not associated
							c.-53G>C, c.-45G>A, c.-63G>C	
Cheng et al., 2012 [35]	Asian, Chinese	Lung cancer	124	126	HB	PCR-RFLP	Ser326Cys	Not associated
Cincin et al., 2012 [36]	Caucasian, Turkish	Endometrial cancer	104	158	HB/PB	PCR-RFLP	Ser326Cys	Associated
Dhillon et al., 2011 [37]	South Australian	Prostate cancer	118	132	HB	PCR-RFLP	Ser326Cys	Associated
Engin et al., 2010 [38]	Caucasian, Turkish	colorectal carcinoma	110	116	HB	PCR-RFLP	Ser326Cys	Not associated
Engin et al., 2011 [39]	Caucasian, Turkish	Gastric cancer	106	116	HB/PB	PCR-RFLP	Ser326Cys	Not associated
Farkasova et al., 2008 [40]	European	Cervical cancer	—	—	HB	PCR-RFLP	Ser326Cys	Not associated
Gangwar et al., 2009 [41]	Asian, North Indian	Urothelial bladder cancer	212	250	HB	PCR-RFLP	Ser326Cys	Associated
García-Quispes et al., 2011 [2]	European, Spanish	Thyroid cancer	402	479	HB/PB	iPLEX	Ser326Cys	Not associated
Jiao et al., 2007 [42]	Asian, Chinese	Gall bladder cancer	204	209	HB	PCR-RFLP	Ser326Cys	Associated
Karahalil et al., 2008 [43]	Caucasian, Turkish	Lung cancer	165	250	HB	PCR-RFLP	Ser326Cys	Not associated
Kim et al., 2013 [44]	Asian, Korean	Breast cancer	346	361	HB	SNP arrays	Ser326Cys	Associated
Kumar et al., 2011 [22]	Asian, Indian	Head and neck cancer	278	278	HB	PCR-RFLP	Ser326Cys	Associated
Laantri et al., 2011 [45]	North African	Nasopharyngeal cancer	598	545	HB	TaqMan assay	Ser326Cys	Not associated
Letkova et al., 2013 [46]	European	Lung cancer	382	379	HB	PCR-RFLP	Ser326Cys	Not associated
Li et al., 2011 [47]	Asian, Chinese	Lung cancer	455	443	HB	PCR-RFLP	Ser326Cys	Associated
Li et al., 2011 [48]	Asian, Chinese	Lymphoblastic leukemia	415	511	HB	TaqMan assay	Ser326Cys	Associated
Li et al., 2013 [49]	Asian, Chinese	Nasopharyngeal cancer	231	300	HB	PCR-RFLP	Ser326Cys	Not associated
Liu et al., 2010 [50]	Asian, Taiwan	Lung cancer	358	716	HB	PCR-RFLP	Ser326Cys	Associated
Luo et al., 2014 [17]	Asian, Chinese	Breast cancer	194	245	HB	PCR-CTPP	Ser326Cys	Not associated
Mahjabeen et al., 2011 [19]	Asian, Pakistani	Head and neck Cancer	300	300	HB/PB	PCR-SSCP	Asp267Asn, Ser279Gly	Associated
							Ile253Phe, Ala399Glu	
Malik et al., 2010 [51]	Asian, Indian	Gastric Cancer	108	195	HB	PCR-RFLP	Ser326Cys	Not associated
Mandal et al., 2012 [52]	Asian, Indian	Prostate cancer	192	224	HB/PB	PCR-RFLP	Ser326Cys	Not associated
Hsu et al., 2010 [53]	Asian, Chinese	Breast cancer	401	533	HB	PCR-RFLP	Ser326Cys	Associated
Mitra et al., 2011 [54]	Asian, Indian	Head and neck cancer	350	225	HB	PCR-RFLP	Ser326Cys	Associated
Mittal et al., 2012 [55]	Asian, Indian	Prostate cancer	195	250	PB	ARMS-PCR	Ser326Cys	Associated
Mittal et al., 2012 [55]	Asian, Indian	Bladder cancer	212	250	PB	ARMS-PCR	Ser326Cys	Associated
Narter et al., 2009 [56]	Caucasian, Turkish	Bladder cancer	83	45	HB	PCR-RFLP	Ser326Cys	Not associated
Ouyang et al., 2013 [57]	Asian, Chinese	lung adenocarcinoma	82	201	HB	PCR-RFLP	Ser326Cys	Not associated
Park et al., 2007 [58]	Asian, Korean	Colorectal cancer	439	676	HB	Sequencing	Ser326Cys	Not associated
Pawlowska et al., 2009 [59]	European, Polish	Laryngeal cancer	253	253	HB	PCR-RFLP	Ser326Cys	Associated
Przybylowska et al., 2013 [60]	Caucasian Polish	Colorectal cancer	182	245	HB/PB	PCR-RFLP	Ser326Cys	Associated
Ramaniuk et al., 2014 [61]	Russian, Belarus	Colorectal	336	370	HB	PCR-RFLP	Ser326Cys	Associated
Reeves et al., 2012 [62]	Australian/Polish	Colorectal cancer	209	215	HB	RT-PCR	Ser326Cys	Not associated
Romanowicz-Makowska et al., 2008 [63]	Caucasian, Polish	Breast cancer	100	106	HB	PCR-RFLP	Ser326Cys	Not associated
Romanowicz-Makowska et al., 2011 [64]	European, Polish	Endometrial cancer	150	150	HB	PCR-RFLP	Ser326Cys	Not associated
de Ruyck et al., 2007 [65]	European, Belgian	Lung cancer	110	110	HB	PCR-RFLP	Ser326Cys	Negatively associated
Sameer et al., 2012 [66]	Asian, Indian	Colorectal cancer	114	200	HB	PCR-RFLP	Ser326Cys	Not associated
Sangrajrang et al., 2008 [67]	Asian, Thai	Breast cancer	507	425	HB	Melting curve analysis	Ser326Cys	Associated
Santonocito et al., 2012 [68]	European, Italian	Melanoma	167	186	HB	PCR-RFLP	Ser326Cys	Not associated
Santos et al., 2012 [69]	Caucasian	Thyroid cancer	109	217	HB	PCR-RFLP	Ser326Cys	Not associated
Sliwinski et al., 2011 [70]	Caucasian Polish	Head and neck cancer	265	280	HB	PCR-RFLP	Ser326Cys	Associated
Sliwinski et al., 2009 [71]	Caucasian, Polish	Colorectal cancer	100	100	HB	PCR-RFLP	Ser326Cys	Associated
Sobczuk et al., 2012 [72]	Caucasian, Polish	Endometrial Cancer	94	14	HB	PCR-RFLP	Ser326Cys	Not associated
Srivastava et al., 2010 [73]	Asian, Indian	Gall bladder cancer	230	230	PB	PCR-RFLP	Ser326Cys	Associated
Srivastava et al., 2009 [74]	Asian, Indian	Gall bladder cancer	173	204	HB	PCR-RFLP	Ser326Cys	Associated
Stanczyk et al., 2011 [75]	Caucasian, Polish	Acute lymphoblastic leukemia	97	131	HB	PCR-RFLP	Ser326Cys	Associated
Sun et al., 2010 [76]	Asian, Chinese	Gastric cancer	73	255	HB	PCR-RFLP	Ser326Cys	Associated
Upadhyay et al., 2010 [77]	Asian, Indian	Esophageal cancer	335	402	HB	PCR-RFLP	Ser326Cys	Not associated
Wang et al., 2011 [78]	Asian, Taiwanese	Urothelial carcinoma	460	540	HB	PCR-RFLP	Ser326Cys	Associated
Xue et al., 2013 [79]	Asian, Chinese	Lung adenocarcinoma	410	410	HB	PCR-RFLP	Ser326Cys	Associated
Yang et al., 2008 [80]	Asian, Chinese	Laryngeal cancer	72	72	HB	PCR-RFLP	Ser326Cys	Associated
Yuan et al., 2012 [81]	Asian, Chinese	Hepatocellular cancer	350	400	HB	PCR-RFLP	Ser326Cys	Associated
Yun et al., 2012 [82]	Asian, Korean	Prostate cancer	266	266	HB	PCR-RFLP	Ser326Cys	Associated
Zhang et al., 2010 [83]	American	Prostate cancer	193	197	HB/PB	Mass spectrometry	Ser326Cys	Associated
Zhao et al., 2011 [84]	Asian, Chinese	Renal cell carcinoma	572	574	HB	TaqMan Assay	Ser326Cys	Associated

**Table 2:** Mutations and their allele frequencies observed in the OGG1 gene in breast cancer patients.

Mutation/exon Chr3 (GRCh37)	Patients		Controls		^a^Odds ratio (95% CI)	^b^ *p* value
	Number	Allele frequency Minor/major	Number	Allele frequency Minor/major		
g.9792260 insert_T Intron 1	34	T 0.09/0.91	07	T 0.35/0.65	3.80 (1.67 to 8.66)	**0.001**
g.9793680G>A Intron 3 (rs55846930)	19	A 0.05/G 0.95	00	A 00/G 1.0	14.65 (1.95 to 109.90)	**0.009**
g.9793748G>A Intron 3	14	A 0.04/G 0.96	00	A 00/G 1.0	10.70 (1.40 to 81.64)	**0.02**
g.9798336T>G Intron 5	10	G 0.03/T 0.97	06	G 0.40/T 0.60	1.25 (0.45 to 3.46)	0.67
g.9798349T>A Intron 5	34	A 0.09/T 0.91	02	A 0.10/T0.90	13.30 (3.18 to 55.70)	**0.0004**
g.9792109delT splice site Intron 1	26	0.07/T 0.93	00	00/T 1.0	20.07 (2.71 to 148.53)	**0.003**
g.9798307T>G splice site Intron 5	16	G 0.04/T 0.96	00	G 00/T 1.0	12.11 (1.60 to 91.70)	**0.01**
g.9798502T>G splice site Intron 6	18	G 0.05/T 0.95	00	G 00/T 1.0	13.68 (1.82 to 102.90)	**0.01**
g.9800972T>G splice site intron 7a	36	G 0.10/T 0.90	00	G 00/T 1.0	28.85 (3.87 to 207.70)	**0.001**
g.9798848G>A 3′UTR	37	A 0.10/G 0.90	00	A 00/G 1.0	29.20 (3.98 to 213.74)	**0.001**
g.9798896T>C 3′UTR	48	C 0.13/T 0.87	03	C 0.15/T 0.85	12.85 (3.97 to 41.56)	**<0.0001**
g.9793544T>G Exon 3, Val159Gly	18	G 0.05/T 0.95	00	G 00/T 1.0	13.68 (1.82 to 102.90)	**0.01**
g.9796483G>A Exon 4, Gly221Arg (TMP_ESP_3_9796483)	22	A 0.06/G 0.94	00	A 00/G 1.0	16.85 (2.26 to 125.53)	**0.005**
g.9798773C>G Exon 6d Ser326Cys (rs1052133) (CM993185)	24	G 0.06/C 0.94	00	G 00/C 1.0	18.45 (2.49 to 136.99)	**0.004**
g.9807669G>A Exon 8, Trp375STOP	17	A 0.05/G 0.95	00	A 00/G 1.0	12.90 (1.71 to 97.28)	**0.01**

OR: odds ratio; CI: confidence interval. ^a^ORs for logistic regression analysis. ^b^ *p* < 0.05, by *χ* ^2^-test for trend.

**Table 3:** Mutations in OGG1 gene in breast cancer patients with conservation score, SIFT score, Align GVGD score, Grantham distance, and Mutation Taster prediction.

Change in nucleotide with its conservation level PhyloP score [−14.1; 6.4]	Change in codon	Amino acid change with its conservation level (up to 13 species)	SIFT score (median)	Align GVGD score (C0–C65)	Grantham dist. (for physicochemical difference b/w amino acids) (0–215)	Mutation Taster (*p* value)
**g.9793544T>G** Highly conserved nucleotide phyloP: **4.97**	**GTG to GGG**	**Val159Gly** Moderately conserved amino acid	Deleterious 0.01 (2.95)	C0 (GV: 197.52-GD: 72.75)	109 (moderate)	Disease causing (*p* = 1.0)

**g.9796483G>A** Moderately conserved nucleotide phyloP: **2.38**	**GGG to AGG**	**Gly221Arg** Moderately conserved amino acid	Tolerated 0.28 (2.95)	C0 (GV: 161.50-GD: 19.25	125 (moderate)	Disease causing (*p* = 0.999)

**g.9798773C>G** Not conserved nucleotide phyloP: **0.28**	**TCC to TGC**	**Ser326Cys** Weakly conserved amino acid	Tolerated 0.19 (2.95)	C0 (GV: 353.86-GD: 0.00)	112 (moderate)	Polymorphism (*p* = 1.0)

**g.9807669G>A** Weakly conserved nucleotide phyloP: **0.12**	**TGG to TGA**	**Trp375STOP** Moderately conserved amino acid	Deleterious 0.01 (2.95)	C0 (GV: 197.52-GD: 72.75)	170 (large) Protein truncation	Disease causing (*p* = 1.0)

PhyloP was used as a conservation score rating the nucleotides from “not conserved” (−14.1) to “highly conserved” (6.4). Align GVGD score: most likely deleterious (C65) to least likely deleterious (C0) GV (Grantham variation) and GD (Grantham deviation). The Grantham distance was used to evaluate physicochemical changes in amino acids (0 = no physicochemical changes; 215 = large changes). In silico predictions were performed using PolyPhen-2 (Polymorphism Phenotyping-2), SIFT (Sorting Intolerant from Tolerant) score: <0.05 deleterious, >0.05 tolerated, and Mutation Taster: disease causing variants (*p* value = 1.0), might not be disease causing (*p* value <0.99).

**Table 4:** Correlation between OGG1 mutations and tumor types, ER/PR status, and HER-2/*neu* status in breast cancer patients.

Mutation/exon Chr3 (GRCh37)	Type of tumor			ER status		PR status		HER-2/*neu *status	
	DCIS	IDC	ILC	−ve	+ve	−ve	+ve	−ve	+ve
	Number (%)	Number (%)	Number (%)	Number (%)	Number (%)	Number (%)	Number (%)	Number (%)	Number (%)
g.9792260 insert_T Intron 1	6 (7.69)	24 (11.11)	4 (10.25)	20 (9.57)	12 (10.62)	28 (13.66)	6 (4.96)	24 (14.46)	9 (7.44)
g.9793680G>A Intron 3 (rs55846930)	9 (11.54)	8 (3.70)	2 (5.13)	10 (4.78)	9 (7.96)	16 (7.8)	2 (1.65)	15 (9.14)	4 (3.3)
g.9793748G>A Intron 3	8 (10.25)	5 (2.31)	1 (2.56)	10 (4.78)	4 (3.35)	5 (2.43)	9 (7.44)	7 (4.27)	7 (5.78)
g.9798336T>G Intron 5	00	6 (2.77)	4 (10.25)	6 (2.87)	3 (2.65)	8 (3.9)	2 (1.65)	5 (3.04)	4 (3.3)
g.9798349T>A Intron 5	8 (10.25)	21 (9.72)	5 (12.82)	18 (8.61)	13 (11.50)	21 (10.24)	13 (10.74)	16 (9.76)	13 (10.74)
g.9792109delT splice site Intron 1	4 (5.13)	16 (7.4)	6 (15.38)	16 (76.55)	10 (10.62)	18 (8.78)	8 (6.61)	17 (10.36)	12 (9.91)
g.9798307T>G splice site Intron 5	6 (7.69)	10 (4.63)	00	9 (4.30)	7 (6.19)	09 (4.39)	6 (4.96)	8 (4.87)	6 (4.96)
g.9798502T>G splice site Intron 6	4 (5.13)	11 (5.09)	3 (7.69)	16 (76.55)	2 (2.21)	9 (4.39)	9 (7.44)	12 (7.32)	6 (4.96)
g.9800972T>G splice site intron 7a	3 (3.85)	28 (12.96)	1 (2.56)	19 (9.09)	13 (11.50)	14 (6.82)	16 (13.22)	13 (7.93)	16 (13.22)
g.9798848G>A 3′UTR	00	30 (13.89)	3 (7.69)	24 (11.48)	13 (11.50)	16 (7.8)	13 (10.74)	16 (9.76)	8 (6.61)
g.9798896T>C 3′UTR	8 (10.25)	17 (4.63)	5 (12.82)	29 (13.87)	14 (12.38)	11 (5.36)	12 (9.91)	15 (9.14)	11 (9.09)
g.9793544T>G Exon 3, Val159Gly	4 (5.13)	14 (6.48)	00	12 (5.65)	6 (5.30)	16 (7.8)	2 (1.65)	3 (1.83)	12 (9.91)
g.9796483G>A Exon 4, Gly221Arg (TMP_ESP_3_9796483)	5 (6.41)	14 (6.48)	2 (5.13)	15 (7.18)	5 (4.42)	10 (4.87)	8 (6.61)	7 (4.27)	6 (4.96)
g.9798773C>G Exon 6d Ser326Cys (rs1052133) (CM993185)	6 (7.69)	15 (6.94)	1 (2.56)	12 (5.65)	8 (7.08)	19 (9.27)	3 (2.48)	6 (3.66)	7 (5.78)
g.9807669G>A Exon 8 Trp375STOP	7 (8.97)	4 (1.85)	2 (5.13)	9 (4.30)	7 (6.19)	5 (2.28)	14 (11.57)	7 (4.27)	6 (4.96)
Correlation^a^	−0.333^∗∗∗^			0.739^∗∗^		−0.155^∗^		0.318	
*p* value^b^	0.0001			0.001		0.01		0.12	

^a^Pearson correlation coefficient; ^b^ *p* value for *χ* ^2^ test; *p* < 0.05 is considered statistically significant; IDC: invasive ductal carcinoma; DCI: ductal carcinoma in situ; ILC: invasive lobular carcinoma; ER: estrogen receptor; PR: progesterone receptor; HER-2/*nue:* human epidermal growth factor receptor 2. ^∗^ *p* < 0.05, ^∗∗^ *p* < 0.01, ^∗∗∗^ *p* < 0.001.

**Table 5:** Association of OGG1 mutations with family history and menopausal age in breast cancer patients in present study.

Mutation/exon Chr3 (GRCh37)	Family history of cancer			Menopause at ≤50 years			Menopause at >50 years		
	Patients (%)	Control (%)	OR (95% CI), *p* value	Patients (%)	Control (%)	OR (95% CI), *p* value	Patients (%)	Control (%)	OR (95% CI), *p* value
g.9792260 ins_T Intron 1	8 (8.2)	02 (40)	3.0 (0.6 to 14.3), 0.1	17 (11.0)	4 (36.4)	3.2 (1.1 to 9.6), **0.04**	5 (11.1)	2 (25)	1.8 (0.3 to 9.4), 0.4
g.9793680G>A Intron 3 (rs55846930)	12 (12.2)	0	9.1 (1.2 to 70.5), **0.03**	11 (7.1)	0	8.2 (1.0 to 64.0), **0.05**	4 (8.8)	0	2.9 (0.3 to 26.1), 0.3
g.9793748G>A Intron 3	5 (5.1)	0	3.7 (0.4 to 32.2), 0.2	10 (6.5)	0	7.4 (0.9 to 58.5), 0.06	4 (8.8)	0	2.9 (0.3 to 26.1), 0.3
g.9798336T>G Intron 5	6 (6.1)	01 (20)	4.5 (0.5 to 37.6), 0.1	08 (5.2)	2 (18.2)	2.9 (0.6 to 13.9), 0.2	2 (4.4)	2 (25)	0.7 (0.1 to 5.1), 0.7
g.9798349T>A Intron 5	6 (6.1)	01 (20)	4.5 (0.5 to 37.6), 0.1	19 (12.3)	01 (9.9)	14.6 (1.9 to 110.5), **0.009**	4 (8.8)	1 (12.5)	2.9 (0.3 to 26.1), 0.3
g.9792109delT splice site Intron 1	4 (4.1)	0	3.0 (0.3 to 26.9), 0.3	13 (8.4)	0	9.8 (1.3 to 75.3), **0.03**	1 (2.2)	0	0.7 (0.04 to 11.5), 0.8
g.9798307T>G splice site Intron 5	2 (2.0)	0	1.5 (0.1 to 16.5), 0.7	06 (5.2)	0	4.4 (0.5 to 36.6), 0.2	4 (8.8)	0	2.9 (0.3 to 26.1), 0.3
g.9798502T>G splice site Intron 6	12 (12.2)	0	9.1 (1.2 to 70.5), **0.03**	06 (5.2)	0	4.4 (0.5 to 36.6), 0.2	3 (6.6)	0	2.2 (0.2 to 20.9), 0.5
g.9800972T>G splice site Intron 7a	0	0	00	16 (10.3)	0	12.2 (1.6 to 92.7), **0.02**	6 (13.2)	0	4.4 (0.5 to 36.6), 0.2
g.9798848G>A 3′UTR	5 (5.1)	0	3.7 (0.4 to 32.2), 0.2	14 (9.0)	0	10.6 (1.4 to 81.1),** 0.02**	3 (6.6)	0	2.2 (0.2 to 20.9), 0.5
g.9798896T>C 3′UTR	9 (9.2)	01 (20)	6.8 (0.8 to 54.0), 0.07	16 (10.3)	01 (9.9)	12.2 (1.6 to 92.7), **0.02**	3 (6.6)	1 (12.5)	2.2 (0.2 to 20.9), 0.5
g.9793544T>G Exon 3, Val159Gly	4 (4.1)	0	3.0 (0.3 to 26.9), 0.3	02 (1.3)	0	1.4 (0.1 to 15.9), 0.8	1 (2.2)	0	0.7 (0.04 to 11.5), 0.8
g.9796483G>A Exon 4, Gly221Arg (TMP_ESP_3_9796483)	2 (2.0)	0	1.5 (0.1 to 16.5), 0.7	04 (2.6)	0	2.9 (0.3 to 26.1), 0.3	2 (4.4)	0	1.4 (0.1 to 15.9), 0.8
g.9798773C>G Exon 6d, Ser326Cys (rs1052133) (CM993185)	19 (19.4)	0	14.6 (2.0 to 109.9), **0.009**	13 (8.4)	0	9.8 (1.3 to 75.3), **0.03**	3 (6.6)	0	2.2 (0.2 to 20.9), 0.5
g.9807669G>A Exon 8 Trp375STOP	4 (4.1)	0	3.0 (0.3 to 26.9), 0.3	08 (5.2)	0	5.9 (0.7 to 47.5), 0.1	1 (2.2)	0	0.7 (0.04 to 11.5), 0.8

OR: odds ratio; CI: confidence interval. ORs for logistic regression analysis. *p* < 0.05, by *χ* ^2^-test for trend.

**Table 6:** Distribution and association of OGG1 mutations with smoking status in breast cancer patients.

Mutation/exon Chr3 (GRCh37)	Patients		
	Smokers (%)	Nonsmokers (%)	OR^a^ (95%CI), ^ b^ *p* value
g.9792260 ins_T Intron 1	4 (4.6)	30 (12.4)	0.34 (0.12 to 1.0), **0.05**
g.9793680G>A Intron 3 (rs55846930)	5 (5.7)	14 (5.8)	1.0 (0.35 to 2.84), 1.0
g.9793748G>A Intron 3	1 (1.1)	13 (5.4)	0.2 (0.03 to 1.59), 0.13
g.9798336T>G Intron 5	2 (2.3)	08 (3.3)	0.7 (0.14 to 3.31), 0.64
g.9798349T>A Intron 5	5 (5.7)	29 (12.0)	0.45 (0.17 to 1.19), 0.1
g.9792109delT splice site Intron 1	14 (16.1)	12 (5.0)	3.67 (1.6 to 8.3), **0.002**
g.9798307T>G Splice site Intron 5	0	16 (6.6)	0.16 (0.02 to 1.26), 0.08
g.9798502T>G Splice site Intron 6	2 (2.3)	16 (6.6)	0.33 (0.07 to 1.48), 0.1
g.9800972T>G Splice site Intron 7a	15 (17.2)	21 (8.7)	2.19 (1.07 to 4.48), **0.03**
g.9798848G>A 3′UTR	4 (4.6)	33 (7.8)	0.3 (0.1 to 0.88), **0.03**
g.9798896T>C 3′UTR	09 (10.3)	39 (16.1)	0.6 (0.28 to 1.3), 0.2
g.9793544T>G Exon 3, Val159Gly	2 (2.3)	16 (6.6)	0.3 (0.07 to 1.47), 0.15
g.9796483G>A Exon 4, Gly221Arg (TMP_ESP_3_9796483)	4 (4.6)	18 (7.4)	0.6 (0.2 to 1.8), 0.3
g.9798773C>G Exon 6d, Ser326Cys (rs1052133) (CM993185)	17 (19.5)	07 (2.9)	8.1 (3.2 to 20.4), **<0.0001**
g.9807669G>A Exon 8 Trp375STOP	3 (3.4)	14 (5.8)	0.6 (0.16 to 2.1), 0.4

^a^OR: odds ratio; CI: confidence interval. ^a^ORs for logistic regression analysis. ^b^ *p* < 0.05 is considered statistically significant, by *χ* ^2^-test for trend.
