# Supplementary material for: OGG1 Mutations and Risk of Female Breast Cancer: Meta-Analysis and Experimental Data
Source: Dis Markers. 2015 May 19;2015:690878. doi: 10.1155/2015/690878 (PMC4452349; doi:10.1155/2015/690878)
Supplement: Supplementary file 1 — In Supplementary material, supplementary Table 1 contained the detailed information regarding the demographic characteristics of patients and controls of sampled population. Significantly increased risk of breast cancer was found associated in patients with family history of cancer and similar trend was found in case age of menopause. Supplementary Table 1 also showed that significantly higher percentage of breast cancer patients were smokers as compared to controls. Supplementary Table 2 contained the information regarding the histopathological parameters of study cohort and majority of breast cancer patients were with invasive ductal carcinoma with grade-II and negative ER, PR and HER-2/neu status. Supplementary Table 3 represents the details of mutations observed in the OGG1 gene in breast cancer patients along with possible consequences of these changes at protein level. [file 690878.f1.pdf]

Supplementary Table 1: Demographic characteristics of patients and controls in present case-control study.

Variables	Patients (N=530)	Control (N=395)	OR (95%CI)	p-value
**1. Age (years)**				
**Mean (±S.D)**	46.4 (±11.59)	42.80 (±12.96)		
**≤40**	188 (35.47%)	163 (41.30%)	0.78 (0.6 to 1.0)	<0.07
**>40**	342 (64.53%)	232 (58.70%)		
2. **Family History**				
**Yes**	98 (18.49%)	19 (4.81%)	4.49 (2.69 to 7.47)	**<0.0001**
**No**	432 (81.51%)	376 (95.19%)		
**3. Age at menarche** (years)				
**<13**	133 (25.09%)	98 (24.81%)	1.01 (0.75 to 1.37)	<0.9
**≥13**	397 (74.91%)	297 (75.19%)		
4. **Menopausal Status**				
**Pre-menopausal**	288 (54.34%)	222 (56.20%)	0.92 (0.71 to 1.20)	<0.5
**Post-menopausal**	242 (45.66%)	173 (43.80%)		
**5. Age at Menopause**				
**≤50 years**	186 (35.09%)	75 (18.99%)	4.34 (2.84 to 6.63)	**<0.0001**
**>50 years**	56 (10.57%)	98 (24.81%)		
**6. Parity**	**(Married Patients 487)**	**(Married Controls 326)**		
**Nulliparity (P0)**	63 (13%)	49 (15.01%)		0.36
**Uni/bi-parity (P1-2)**	129 (26%)	81 (24.85%)		
**Multiparity (P3-n)**	295 (61%)	196 (60.12%)		
**7. Age at First Birth**	**(total=424)**	**(total=277)**		
**< 25 years**	299 (70.52%)	179 (64.62%)		0.1
**≥ 25 years**	125 (29.48%)	98 (35.38%)		
**9. Smoking History (cigarette, Pan, Chhalia, Naswar,)**				
**Non-Smokers**	443 (84%)	351(88.86%)		**0.03**
**Smokers**	87(16%)	44 (11.14%)		

***OR, odds ratio; CI, confidence interval. ORs for logistic regression analysis.**

p>0.05, by χ^2 -test for trend

Supplementary Table 2: Histopathological characteristics of cancer patients in present case-control study.

1. Site of breast tumor (N= 530)			
**Variables**	**Bilateral**	**Unilateral Right**	**Unilateral Left**
**No (%age)**	26 (4.9%)	231(43.6%)	273(51.5%)

2. Tumor Type (N=333)			
**Variables**	**DCI**	**IDC**	**ILC**
**No (%age)**	78 (23.42%)	216 (64.86%)	39 (11.71%)

3. Tumor Grade (N= 447)				
**Variables**	**Grade I**	**Grade II**	**Grade III**	**Grade IV**
**No (%age)**	110 (24.61%)	212 (47.43%)	112 (25.05%)	13 (2.90%)

4. ER Status (N=322)		
**Variables**	**ER-ve**	**ER+ve**
**No (%age)**	209 (64.9%)	113 (35.1%)

5. PR Status (N=326)		
**Variables**	**PR-ve**	**PR+ve**
**No (%age)**	205 (62.88%)	121(37.12%)

6. HER-2/*neu* status (N=285)		
**Variables**	**HER-2/*neu* -ve**	**HER-2/*neu* +ve**
**No (%age)**	164 (57.54%)	121 (42.46%)

DCI, Dutal Carcinoma in Situ; IDC, Invasive Ductal Carcinoma; ILC, Invasive Lobular Carcinoma;
ER, Estrogen Receptor; PR, Progesterone Receptor; HER-2/*neu*, Human Epidermal growth factor Receptor *2*

Supplementary Table: 3 Mutations observed in the OGG1 gene in breast cancer patients

MUTATION/ EXON	Change at Genomic DNA Chr3(GRCh37)	Change at cDNA Level NM_002542.5	Change at Protein Level/ possible consequence
**Insertion/ Intron 1**	g.9792260ins_T	c.137+153dupT	
**Substitution/ Intron 3** (rs55846930)	g.9793680G>A	c.565+47G>A	
**Substitution/ Intron 3**	g.9793748G>A	c.565+115G>A	
**Substitution/ Intron 5**	g.9798336T>G	c.898+31T>G	
**Substitution/Intron 5**	g.9798349T>A	c.898+44T>A	
**Splice site deletion/ Intron 1**	g.9792109del T	c.137+2delT	Variation in donor splice site 2 bps upstream & skip of exon 1 is very likely
**Splice site substitution / Intron 5**	g.9798307T>G	c.898+2T>G	Variation in donor splice site 2 bps upstream & skip of exon 5 is very likely
**Splice site substitution / Intron 6**	g.9798502T>G	c.948+2T>G	Variation in donor splice site 2 bps upstream & skip of exon 6 is very likely
**Splice site substitution / Intron 7a**	g.9800972T>G	c.1048+2T>G NM_016828.2	Variation in donor splice site 2 bps upstream & skip of exon 7a is very likely
**Substitution/ 3' UTR**	g.9798848G>A	c.*14G>A	
**Substitution/ 3' UTR**	g.9798896T>C	c.*62T>C	
**Missense/ Exon 3**	g.9793544T>G	c.476T>G NM_016821.2	**p.Val159Gly** variant in protein domains HhH-GPD & 8-oxoguanine DNA-glycosylase
**Missense/ Exon 4** (TMP_ESP_3_9796483)	g.9796483G>A	c.661G>A NM_016821.2	**p.Gly221Arg** variant in protein domains HhH-GPD & 8-oxoguanine DNA-glycosylase
**Missense/ Exon 6d** (rs1052133)(CM993185)	g.9798773C>G	c.977C>G	**p.Ser326Cys** variant is in protein domain: 8-oxoguanine DNA-glycosylase
**Termination/ Exon 8**	g.9807669G>A	c.1125G>A NM_016821.2	**p.Trp375STOP** Premature termination resulted in truncated protein

rs = reference single nucleotide polymorphism (SNP) identifier; Nomenclature of sequence variants follows the recommendations by the Human Genome Variation Society (HGVS, http://www.hgvs.org/mutnomen Classification of novel sequence variants. HhH-GPD= hallmark helix-hairpin-helix and Gly/Pro rich loop domain.
